# Low provider and staff self-care in a large safety-net HIV clinic in the Southern United States: Implications for the adoption of trauma-informed care

**DOI:** 10.1177/2050312119871417

**Published:** 2019-08-21

**Authors:** Jessica M Sales, Kaitlin Piper, Clara Riddick, Betelihem Getachew, Jonathan Colasanti, Ameeta Kalokhe

**Affiliations:** 1 Department of Behavioral Sciences and Health Education, Rollins School of Public Health, Emory University, Atlanta, GA, USA; 2 Department of Global Health, Rollins School of Public Health, Emory University, Atlanta, GA, USA; 3 Division of Infectious Diseases, School of Medicine, Emory University, Atlanta, GA, USA

**Keywords:** Self-care, burnout, trauma-informed care, HIV/AIDS

## Abstract

**Objective:**

This mixed-methods needs assessment study examined self-care practices among providers, staff, and administrators at an HIV clinic and identified barriers and facilitators to strengthening self-care services in this setting.

**Methods:**

Surveys (n = 31) and qualitative interviews (n = 23) were conducted with staff, providers, and administrators at a large, safety-net HIV clinic.

**Results:**

Surveys indicated an overall absence of formal self-care services including resources to manage stress, opportunities to debrief, and formal mechanisms to voice concerns. Based on interviews with staff and providers, deficiencies in self-care services included support for dealing with complex patients, formal mechanisms for feedback, and time for self-care. Administrators recognized the need for more support, acknowledged that opportunities for employees to voice concerns were lacking, and felt that implementing multi-disciplinary team meetings could improve morale and reduce stress and burnout.

**Conclusion:**

This assessment revealed a need to enhance self-care in safety-net HIV services. Adoption of trauma-informed care, which includes activities to strengthen self-care, could reduce workplace burnout.

## Introduction

To provide effective patient care, healthcare professionals must, among other things, maintain their own well-being.^1–5^ However, high healthcare system demands have made this imperative particularly difficult. The healthcare environment—with high patient volumes, limited time, and numerous administrative and clinical responsibilities—places physicians and other healthcare professionals at risk for burnout.^1–4^ Burnout is not only a function of exhaustion, overwork, and stress^5,6^ but also a complex psychological and sociological condition that leads to depersonalization (detached feelings toward clients or patients), loss of caring, and lack of engagement, which can be detrimental to therapeutic environments and patient health outcomes.^3,7,8^ Burnout can also result in a reduced sense of personal accomplishment and satisfaction with job performance, negatively impacting organizational morale, productivity, and employee retention.^5,9,10^

Studies report over half of physicians in the United States experience symptoms of burnout,^3,11,12^ which is nearly double the prevalence among the general working population.^11,13^ Also, clinician burnout has substantially increased in recent years, rising approximately 9% between 2011 and 2014, while the prevalence of burnout remained stable among other professions.^13^ Major drivers of burnout relate to the growing productivity demands of the healthcare system and profuse regulatory requirements related to billing, quality, safety, and compliance that decrease face-to-face time with patients.^5^ Among a sample of 422 providers, one study found that approximately 50% reported high levels of work-related stress, frequently encountered time-pressures during patient exam, and felt that their work environment was chaotic.^1^ One-third of the providers intended to leave the practice in the next 2 years.^1^ Although there are increasing rates of burnout among clinicians and growing healthcare demands, implementation of prevention interventions is lacking among healthcare organizations. Based on one survey of 350 clinicians, 66% of respondents felt that they did not have the tools or resources to handle burnout, and 54% said administrators and leaders were not actively taking steps to prevent burnout.^12^

In the context of caring for people living with HIV (PLWH), multi-disciplinary healthcare professionals are involved in the provision of care including physicians, nurses, medical assistants, psychologists, social workers, and other support services (e.g. health educators and peer counselors). Many healthcare professionals serving PLWH, particularly in the early years of the HIV epidemic (the late 1980s and 1990s), reported that working with PLWH was physically and emotionally difficult at times.^14,15^ Furthermore, the caregiving needed to provide for the complex, multi-dimensional needs of PLWH has been linked to work overload, anxiety/psychological stress, and burnout in several studies.^15–18^ More recent studies, after the discovery and wide-scale availability of highly effective antiretroviral therapy for HIV treatment, among healthcare professionals working in the context of HIV care, continue to find high levels of burnout among various healthcare professionals,^19–22^ with one study among HIV community nurses reporting that 66% of nurses experiencing moderate or high levels of burnout according to the Maslach Burnout Inventory (MBI),^19^ and another conducted with HIV physicians and nurses reporting that 76.9% met the MBI criteria for burnout.^20^

Provider and staff burnout can have many negative consequences for healthcare professionals, patients, and organizations. Among healthcare professionals (ranging across professions-physicians, nurses, and other staff professionals), not only is burnout associated with a loss of fulfillment, decreased job satisfaction, and increased workloads^13,23^ but is also linked to mental health disorders, depression, and suicide.^5^ Approximately 300–400 practicing providers die of suicide each year,^24^ which is twice the rate of suicide among the general population.^25^ Burnout also threatens the health of patients, through loss of face-to-face time with clinicians, delays in access to care, and suboptimal health outcomes.^3,5^ It is also associated with an increased risk for medical errors and malpractice, lower rates of patient treatment adherence, and decreased patient satisfaction.^3,5^ Furthermore, healthcare organizations are negatively impacted, as burnout-induced annual turnover expenses can amount to over US$40 million per health system.^26–28^ These organizations may also incur excess costs due to productivity declines and malpractice-related expenses.^26^ Costs to society are also large; a recent study in Canada estimated that burnout among a cohort of 70,000 physicians would cost the country US$213 million in lost health services over a 24-year period, based on reductions in clinic hours by burnt-out physicians and increased early retirement.^29^

For healthcare organizations treating patients with trauma histories, it can be especially challenging to reduce burnout and promote provider and staff well-being.^30^ Although literature is limited among providers and staff working in environments serving high numbers of patients with trauma histories, such as mental health facilities or safety-net clinics, including safety-net HIV clinics, evidence suggests there is an increased risk of emotional exhaustion, stress, and burnout among clinicians that are repeatedly exposed to patients with complex trauma histories.^8,31^ These professionals may even experience secondary trauma or vicarious trauma, exhibiting symptoms of acute distress or post-traumatic stress ^4,7^. Because of the risk of burnout among providers working in settings serving patients with trauma histories, trauma-informed care (TIC) models encourage provider and staff self-care practices.^30^ As a central tenant of TIC, self-care is an evidence-based, essential practice for professionals working with traumatized patients to address associated secondary trauma and burnout.^30^ To promote the well-being of healthcare professionals, it is recommended that organizations, especially those that treat populations with high rates of trauma, have procedures to help staff manage stress and emotional fatigue.^6,30,32^ Organizational TIC self-care practices would ideally include ongoing trauma training, opportunities to debrief after a crisis or difficult patient, peer support, and resources to manage stress responses.^6,30,32^

At safety-net clinics, patients often have complex trauma histories along with other social and economic challenges, such as poverty, low literacy, and lack of insurance.^33^ Not only are healthcare professionals working at these clinics exposed to complex and medically vulnerable populations but they also often face additional stressors due to lack of essential supplies and resources, limited patient care space, and understaffing.^9,34^ Because these resource-poor environments place medical staff at risk for burnout,^9,34^ it may be necessary for safety-net clinics to provide self-care and support services. Furthermore, because rates of trauma and other psychosocial needs are especially high among persons living with HIV, thereby complicating their treatment engagement and mental and physical health outcomes,^35^ it may be particularly necessary for publicly funded HIV treatment centers that serve as long-term primary care homes to ensure staff and providers are receiving support and self-care services. However, very little is known about the availability of self-care or support services in the context of service provision in publicly funded HIV clinics, or the barriers and facilitators to adopting self-care and support services in HIV care settings. Through a mixed-methods needs assessment study, we aimed to understand self-care practices and services at a large, urban safety-net HIV center that serves a largely uninsured population in the South.

## Methods

### Ethics statement

The study was approved by the Emory University Institutional Review Board and Grady Research Oversight Committee (IRB00090994). All potential participants provided written informed consent prior to engaging in study activities.

### Study setting and context

The study was conducted at an urban, HIV care center in the Southern United States that serves over 6000 persons living with HIV. The South is home to 45% of the country’s individuals living with HIV and 50% of the country’s new HIV infections, rendering it the current epicenter of the US HIV epidemic.^36^ The care center, one of the largest Ryan White-funded centers in the United States, serves a diverse population of adults, young adults and adolescents, and children. Approximately, three-quarters are men, one-quarter women, and <1% transgender. The majority are African American (83% or 4703), 10% (593) are White, and 5% (281) are Hispanic. The most common risk factors for acquisition of HIV are men having sex with men (46% or 2609), heterosexual contact (40% or 2267), perinatal transmission (8% or 462), and injection drug use (3% or 169). Patients come from diverse socioeconomic backgrounds, however the majority are low-income, live in poverty, and are un- or under-insured. In addition to facing socioeconomic stress, many have histories of trauma, substance abuse, homelessness, and transactional sex, and suffer from stigma, discrimination, shame, comorbid disease, and poor mental health.^37^ Thus, the complex psychosocial and medical needs of many of the patients served in this high-volume HIV care center contribute to the stress and workload of providers/staff. The center is staffed by 160 individuals representing various professional roles including physicians, nurse practitioners, physician assistants, nurses, social workers, case managers, nursing/medical assistants, laboratory technicians, counselors, security personnel, and clinic clerks. The center is comprised of four clinics (main clinic, serves HIV primary care needs of men; women’s clinic, serves the primary HIV care needs of women; pediatric/adolescent clinic serves HIV care needs of youth; and mental health clinic, services mental health needs of all patients), as well as onsite dental, legal, housing, child care, pastoral services, substance abuse treatment, case management, and peer counseling.

### Study design

Between March 2017 and January 2018, we conducted a comprehensive multi-tier needs assessment employing a mixed-methods convergent parallel design. A convergent parallel design weights both qualitative and quantitative methods equally, and concurrently collects quantitative and qualitative data in the same time of the research process. The two components are analyzed independently, but the results of both are interpreted.^38^ As part of the greater needs assessment, we conducted quantitative assessments and in-depth interviews with HIV care providers and staff at the center. We also conducted in-depth interviews with center administrators.

### Participant recruitment

Prior to initiation of research activities, the team met with key center administrative stakeholders to introduce and assess support for the study, address questions on the study and potential implications of findings, and to obtain feedback on the study design and recruitment procedures. All providers and staff were invited to participate in the quantitative assessment. We used purposive sampling for the in-depth interviews to sample providers and staff to ensure adequate representation from the different clinics and services within the center and roles of staff. We defined providers as physicians, nurse practitioners, and physician assistants who provide HIV clinical care. Staff were defined as nurses, social workers, case managers, nursing assistants, laboratory technicians, counselors, security personnel, and clinic clerks. Providers and staff were recruited by email invitation and flyers posted throughout the center. Center administrators were invited by email to participate in an in-depth interview.

### Data collection

After learning about the focus of the study and providing informed consent, staff and providers were given the option of completing the quantitative assessment and/or in-depth interview, and administrators were given the opportunity to participate in an in-depth interview. The quantitative assessments were self-administered via SurveyGizmo and completed by the participants on their clinic or personal computers. Research assistants were available to provide technical support. In-depth interviews were conducted by female Master’s-level qualitatively trained study team members, including co-author (C.R.). Participants received US$25 for completing the quantitative assessment and US$50 for completing the in-depth interview.

The quantitative assessment tool was adapted from the National Center on Family Homelessness “Trauma-informed Organizational Toolkit,” which was designed to help centers evaluate their current practices and transition to a TIC model to better support healing and recovery of clients, as well as address burnout and stress among providers and staff.^39^ Adaptation involved review of the tools by HIV researchers and providers practicing at the center, behavioral scientists, and local TIC experts in the context of services available at the center or the surrounding community, followed by editing by the research team to enhance readability. The quantitative assessment spanned five implementation domains identified as essential to TIC delivery:^40^ (1) training and workforce development; (2) physical environment; (3) screening, assessment, and treatment services; (4) engagement and involvement; and (5) cross-sector collaboration. However, this article solely focuses on the first domain: training and workforce development. Seventeen closed-ended items in the provider/staff assessment for this domain surveyed center practices regarding self-care. Example survey items included “self-care is supported and encouraged by policy and practice at the Center” and “the Center helps staff members debrief after a crisis.” Answer options ranged from “strongly disagree” to “strongly agree.” Three open-ended questions followed to gain a more in-depth understanding of participant perspectives about self-care/support and facilitators and barriers.

In-depth interviews were conducted in person by female Master’s-level qualitatively trained study team members (including author C.R.), in a private space in the clinic. Average interview duration was 30–60 min. The interview guide was adapted from the “Creating Cultures of Trauma-informed Care” materials^41^ and explored several TIC implementation domains; however, only the themes related to self-care and support services are presented here. Questions for this domain explored current center practices, capacity, weaknesses, and strengths in promoting self-care and supportive environments for staff and providers. Example questions included: “What are the strengths of the Center in this [self-care and supporting needs of staff] area? How could things be improved?” All interviews were audio recorded and transcribed verbatim.

### Data analysis

A 4-point Likert-type scale was applied to the quantitative assessment responses (i.e. “strongly disagree”= 0, “disagree”= 1, “agree”= 2, and “strongly agree”= 3). The distribution of responses for each item was examined. Then, the average for each item was calculated (excluding the “I don’t know” responses). To guide interpretation of averages, per scoring recommendations,^39^ items with scores ⩾2 suggested there was consensus on the availability of self-care services. Qualitative data were analyzed using QSR NVivo qualitative software^42^ and thematic analyses. Three members of the study team (Master’s-level graduate research assistants trained in qualitative coding) independently reviewed 20% of transcripts, which they used to generate preliminary codes and code definitions using inductive and deductive methods.^43^ This process continued until saturation of codes (when no team member identified new codes) was reached. Once the codebook was developed, five transcripts were selected for inter-coder agreement exercises. After independently coding the five transcripts, coders met to discuss their analysis and conflicts were resolved by consensus between the three coders. After consensus was achieved, each coder was then responsible for coding approximately one-third of the remaining transcripts. The full study team met bi-weekly during coding to review themes as they evolved. Major themes, across all participants, were then consolidated into a narrative, and when indicated, analyzed separately by role (e.g. staff, provider, and administrator).

## Results

In total, 14 providers and 17 staff completed quantitative assessments, and 9 providers, 10 staff, and 4 administrators completed in-depth interviews. Providers included physicians and advanced practice providers; staff included social workers, case managers, patient navigators, health educators, peer counselors, nurses, translators, patient access representatives, and pastoral and palliative care providers. Providers and staff from all four clinics and key center support services (i.e. social services, pastoral care, and education) participated. Administrators included those who worked with staff and providers, as well as those who were part of the executive leadership of the center.

### Quantitative surveys

Providers had a mean score of <2 on 16 of 17 survey items and staff had a mean score of <2 on 11 of 17 survey items, indicating strong consensus about the overall absence of self-care/clinic support services at the center. Provider and staff self-care services that were absent from the center included: meetings that address trauma and self-care, supervision and training from trauma experts, presence of administrators that understand the emotional impact on staff/providers, spaces to debrief after a crisis, and resources that help staff/providers manage stress reactions. For providers, regular team meetings were the only source of support available. However, staff indicated availability of additional clinic support services, which included policies and procedures that support staff self-care, adequate support in dealing with challenging client situations, supervisors that value their opinion, presence of a formal system for reviewing staff performance, and the opportunity for ongoing evaluation of clinic operations. See Table 1 for a complete list of providers’ and staffs’ mean scores across the 17 survey items.

**Table 1.** Provider and staff survey responses.

Self-care and clinic support items^a^	Providers (n = 14)					Staff (n = 17)				
	Strongly disagree	Disagree	Agree	Strongly agree	Mean	Strongly disagree	Disagree	Agree	Strongly agree	Mean
Staff^b^ members have regular team meetings.	0	1	8	5	2.3	0	1	7	9	2.5
Topics related to trauma are addressed in team meetings.	1	6	3	0	1.2	1	8	3	4	1.6
Topics related to self-care are addressed in team meetings (i.e. vicarious trauma, burnout, and stress-reducing strategies)	1	8	2	0	1.1	0	7	6	3	1.8
Self-care is encouraged and supported with policy and practice at the Center.	1	8	3	0	1.2	0	5	7	5	2.0
Staff members meet with their supervisor/director regularly.	1	3	8	0	1.6	1	7	8	0	1.4
Staff members receive individual supervision from someone who is trained in understanding trauma.	4	8	0	0	0.7	1	6	1	4	1.7
Part of staff’s time with their supervisor/director is used to help staff members understand their own stress reactions.	4	6	1	0	0.7	2	6	1	5	1.6
Part of the staff’s time with their supervisor/director is used to help staff members understand how their stress reactions impact their work with patients.	3	7	1	0	0.8	1	5	3	5	1.9
The Center helps staff members debrief after a crisis.	1	7	2	1	1.3	3	3	1	5	1.7
The Center has a formal system for reviewing staff performance.	2	0	8	3	1.9	0	0	8	9	2.5
The Center provides opportunities for ongoing staff evaluation of the program.	2	6	4	1	1.3	1	0	9	6	2.3
Staff have adequate support in dealing with challenging client situations.	2	4	7	1	1.5	2	3	3	8	2.1
Supervisors have an understanding of the emotional impact (burnout, vicarious trauma, and compassion fatigue) associated with their work.	2	1	8	0	1.5	2	4	5	4	1.7
The Center provides opportunities for staff input into program practices.	3	4	7	0	1.3	2	4	7	4	1.8
The actions that follow (solicitation of input) demonstrate that staff have been heard.	5	6	1	0	0.7	2	4	6	5	1.8
Supervisors communicate that staff members’ opinions are valued even if they are not always implemented.	4	3	6	0	1.2	1	2	9	5	2.1
Outside consultants with expertise in trauma provide ongoing education and consultation.	5	6	0	0	0.5	2	4	2	6	1.9

Shading indicates the mean is ⩾2, suggesting availability of self-care/support service.

a Answer options ranged from 0 = strongly disagree to 3 = strongly agree.

b The term “staff” in the quantitative assessments was defined as inclusive of both center providers and staff. Participants were provided this definition as part of the introduction to the assessment.

### Qualitative interviews

Below we discuss the six prominent themes from interviews with staff, providers, and administrators. Themes and descriptions can be found in Table 2.

**Table 2.** Qualitative themes and descriptions.

Theme	Description	Example quote
Stress and burnout	Stressors associated with caring for patients with complex needs (i.e. compassion fatigue). This also includes stressors associated with working in low-resource, high-demand environments.	We are trying to cover like 800 patients at this moment with whatever [staff] we are left with. (Provider).
Experiences of trauma	Trauma experienced by providers and staff. This can include trauma resulting from interactions with traumatized patients (i.e. secondary trauma).	Having personally experienced trauma in the recent years, I feel I am much more aware of signs and symptoms of trauma among my patients. (Staff)
Peer support	How peer interactions and team meetings help/do not help staff and providers deal with stress, burnout, and secondary trauma.	We are all supportive of one another regarding venting and dealing with our own trauma and the emotional toll of dealing with traumatized patients. (Provider)
Supervisor support	How supervisors help/do not help staff and providers deal with stress, burnout, and secondary trauma.	If I did have a question or concern about trauma, I would be able to address my concerns with my coworkers and supervisor. (Staff)
Institutional support	How policy and services at the organizational-level help/do not help to promote self-care practices among staff and providers.	A self-care class or training would be very beneficial to all staff. (Staff)
Feedback mechanisms	Formal and informal mechanisms through which Center administrators receive feedback on staff and provider needs as well as mechanisms through which administration addresses feedback.	I think it still seems like pretty much a one way street. I mean, I’ve tried to foster a culture of, you know, administration also engaging in discussions around what’s feasible and realistic in terms of change that’s requested, wanted, desired, demanded for the staff and providers. (Provider)

#### Stress and burnout

Caring for patients with complex needs, especially in resource-constrained environments, placed the participants at risk for burnout:

Working for public health institutions is stressful. Working for academic institutions is stressful. Working for grant-funded institutions is stressful. Taking care of people who have this really challenging disease [HIV] who have a huge amount of social needs and poor health literacy is extremely stressful. And so when we have all of those things come together, like we’re all just like in a little rat’s nest, and I mean obviously I care about what I do here because I’ve been here a long time and I don’t want to change my relationship with working in this institution, but it’s extremely stressful. (Provider)

Compounding stress levels, providers and staff believed there were not enough staff to handle the high patient volume. A staff member described the low provider-to-patient ratio in her department: “We have five people that are gonna cover almost 5,000 [patients] in the building” (Staff). The volume of patients needing treatment left providers and staff with minimal time to spend on self-care:

With our lack of providers. . .We are stretched to the max. Like each of my slots is double-booked. That means that half-hour [break] that I need desperately, I have to cut it down, because there’s too many patients who don’t have a provider. (Provider)

Administrators were also aware of the burden placed on providers and staff at the Center:

I will say just very honestly, I think as a clinic in general we are bursting at the seams in terms of our volume and our numbers of patients, and our lack of human resources. . .I think most people in this building feel like they’re overworked. (Administrator)

Participants believed issues related to stress and burnout were common among healthcare workers across the United States: “[Providers] are just being beaten up, but health care is not easy. It’s not just [the Center]. This is health care in the United States” (Staff). One common suggestion to decrease the workload was to transfer some provider responsibilities, such as clerical duties and patient referrals, to medical assistants and support staff:

So we have discussed trying to hire a dedicated person to handle all referrals. It’d be like a medical assistant, and I think that would really help providers and nursing staff in terms of the workload, because, to be honest, there’s at least one or two referrals generating per patient. (Provider)

#### Experiences of trauma

In addition to experiencing stress and burnout, many providers and staff had histories of trauma: “Staff may have had trauma in their lives and they don’t even understand themselves how to address their own personal trauma” (Staff). Without having the tools necessary to manage their mental health needs, not only was trauma impacting providers and staff’s own well-being, but it was also impacting the quality of care they could deliver to patients: “I definitely think that there’s staff that have a reason why they don’t deal with the clients well because they have their own [trauma] still going on” (Staff). Administrators were also aware of the risk of secondary trauma, yet recognized the lack of self-care opportunities for staff:

[Difficult patient interactions] can actually be kinda traumatic to providers in and of themselves. We don’t do a very good job of recognizing that this job can actually often be very stressful on the providers, and they don’t have any outlet or feel supported in any way. (Administrator)

#### Peer support

Participants often described the Center as a “family” and noted that their primary source of emotional support arose from peer interactions and team gatherings:

Staff members often come together and have lunches, parties, and other informal activities that become ways for staff to cope with the stressors of working with a challenging population. In lieu of formal self-care opportunities, the staff have inadvertently found ways to share together, vent together, and take care of one another so that we can best take care of patients. (Provider)

Participants also discussed how multi-disciplinary team meetings provided opportunities for staff to debrief and work together to address concerns with challenging patients:

[Providers] really like having opportunities to discuss how hard their days are or how hard their work is. So the most success I’ve had in terms of engaging providers and them walking out of the meeting feeling good is when we’ve had multidisciplinary team meetings, where we would discuss cases that were challenging. So we were essentially a group venting and debriefing to each other, but I think that was probably one of the most bonding experiences that I’ve experienced. (Provider)

Another provider discussed how working within a multi-disciplinary team enabled them to improve clinic operations and effectively engage with administrators:

I work with a multidisciplinary group within the clinic that helps to manage HIV infected pregnant women, and we specifically, as part of discussions in that group, identified several clinic processes that were not working for our population, and when those were brought up specifically to various members of administration, some of those hurdles were removed and the processes were worked out. (Provider)

Administrators were also hopeful that multi-disciplinary meetings would help reduce burnout and stress among providers and staff at the Center:

And I think they just really enjoyed that aspect to be able to talk about these [difficult patient interactions]. . .They certainly like anything that kind of makes them feel like they have a little support to deliver the care they need to the patients. . . it just goes back to feeling supported and feeling like they’re not on an island by themselves trying to take care of these incredibly complex patients. (Administrator)

Although informal peer support was available at the Center, providers and staff wanted more opportunities to share their experiences with trauma and burnout, perhaps through increasing the frequency of multi-disciplinary team meetings or ensuring an intentional focus on the experiences of burnout at these meetings:

I think it would be useful to hear about experiences of other staff members who have successfully assisted their patients with different types of trauma management in order to learn about experiences that staff have gone through in taking care of these patients and how they coped with the emotional difficulties of working with specific instances and overall with a population at high risk of trauma. (Provider)

Ultimately, participants felt that peer support alone was ineffective at mitigating the high levels of trauma and burnout experienced by staff and providers at the Center: “Peer support and commitment to one’s team members is significant, but it is not enough to sustain this difficult work” (Provider).

#### Supervisor support

In addition to peer support, supervisors tried to foster an environment that was responsive to the needs of providers and staff:

[My supervisor] sits down with each one of us, asks us what do you need for your job, what are your concerns, how are you? They consider our well-being, our own mental health as an important part of our job, especially when we hear about so much trauma that occurs. I have a lot on my case load, so she even encourages us to take mental health days. (Staff)

Although supervisors made every effort to address staff concerns and funnel issues to higher level administrators, they felt that the demands of the Center prevented them from adequately attending to staff needs:

As a supervisor myself, I do my best to support my fellow providers, but it has been increasingly difficult to protect time for debriefing and self-care sessions due to the demands of the health system. (Staff)

#### Institutional support

Despite support from peers and supervisors, many participants expressed a lack of center-wide formal self-care services:

Staff support around dealing with difficult patients is lacking wholly. . .I think admin/management at a higher level need to be engaged in conversations around patient care as I have often found them to lack understanding and appreciation of what we experience as providers who take care of patients who have, and continue to suffer, traumatic events in their lives. (Staff)

Overall, providers acknowledged the presence of wellness activities, but voiced wanting more formal opportunities for self-care and support, such as peer support groups, systematic opportunities to debrief after crises, and staff training and education:

The Center provides a lot of wellness activities for patients and staff members. . .This can be improved by first recognizing that these events are really self-care opportunities and designing a few of these events for that specific purpose, providing formal ways for people to learn about self-care, trauma, etcetera. (Provider)

In addition to the prevention of burnout and secondary trauma, participants suggested that the Center should provide opportunities for staff and providers to seek treatment for mental health concerns:

I think there should be something also that can help, where we offer the service and it can be done on the job. You could have a counselor on your job to talk about your own situations. I think would be very important. I think it would be beneficial to the clients, cause when a person has dealt with their own trauma they can help the others with their trauma. (Staff)

Because providers have typically been the focus of self-care services, staff emphasized that the Center needs to recognize the risk for burnout and secondary trauma among all healthcare workers:

Clinical staff members sometimes get self-care training as part of their continuing education and as part of the medical, mental health, and/or nursing training they received via their clinical training, but this is not true for all staff members. Many non-clinical staff members interact in meaningful ways with patients and have little to no trauma training. It would be helpful if there were more formal and informal opportunities for staff members to participate in self-care activities and much more recognition for the potential for vicarious trauma. Also, we need a lot more recognition for the potential for staff burnout and compassion fatigue. (Staff)

Ultimately, staff and providers believed that implementing self-care and support services at the Center would reduce burnout and stress, which would—in turn—improve patient care and health outcomes.

#### Feedback mechanisms

Although providers and staff wanted more opportunities for self-care and Center support services, they believed their concerns were not acknowledged or heard by the larger healthcare system leadership (i.e. individuals external to the onsite Center administrators). Staff and providers believed these higher level administrators were unable to empathize with their experiences, especially regarding interactions with complex patients: “We encouraged people to voice their concerns, but I think it’s still very strained and there’s definitely a disconnect between administration, leadership, and providers, and staff” (Staff). Also, as many providers are not directly employed by the Center (but rather provide a contracted service), they do not participate in the Center’s annual satisfaction surveys, through which other Center staff have the opportunity to provide formal feedback regarding self-care, support needs, clinic services and operations.

Center administrators also recognized the disconnect between staff/providers and leadership. They noted that their process for receiving and responding to feedback from providers and staff could be improved: “[Feedback] doesn’t seem to happen very frequently, and often times I think the providers feel a little abstract and disconnected, and there’s not enough assessment of what’s working, what’s not, on a day-to-day level” (Administrator). In addition, even when opportunities to provide feedback are available, staff and providers may feel uncomfortable voicing their concerns, which can be a barrier to administration’s awareness of issues within the Center:

We have staff meetings, all different kinds of meetings where there are opportunities to provide feedback, and again, it’s incumbent upon the staff person to do that. Sometimes you’ve gotta be bold and say, “Hey.” Figure out the way to say it and share your feedback. (Administrator)

Although administrators strived to address all employee concerns, they were unable to make all suggested changes due to competing priorities and resource limitations:

I think that the leadership team tries to respond to staff questions, concerns, needs as best we can. I think there are always opportunities to do better at that, and sometimes it’s about kind of weighing out what are the priority issues, ‘cause 160 people who are in here, everybody got their own issues that they would bring to the table, so some of it is making some decisions about prioritizing. What are the things that are most concerning, that would have the biggest impact if we improved upon that, are impacting most people, that are impacting our patients? So trying to tackle the issues that are gonna have the biggest impact on patients and staff. (Administrator)

## Discussion

Both surveys and interviews revealed a lack of formal self-care services for staff and providers at a large, urban safety-net HIV center. Out of the 17 self-care and support services that were included in the survey, 16 services were absent for providers and 11 were absent for staff. The only support service for which there was consensus regarding availability among the providers was regular team meetings. Staff indicated the availability of additional services including support from supervisors as well as opportunities to provide formal feedback on clinic operations. However, both providers and staff lacked key formal self-care services including trauma training, systematic opportunities to debrief after difficult patients, and stress management support.

Qualitative interviews revealed that both providers and staff loved their jobs and were devoted to the HIV center and its patients; however, they felt burned-out, stressed, and overworked. They wanted the center to provide more self-care services and training on how to manage stress, burnout, and patients with trauma histories. Yet, providers and staff simultaneously acknowledged how clinic time constraints, high patient volumes, and lack of resources were barriers to implementation of self-care services at the center. In addition, disconnects between administrators and employees as well as limited opportunities for providers/staff to voice their concerns prevented center leadership from understanding staff and providers’ self-care needs. However, administrators, providers, and staff all noted that promising support practices such as multi-disciplinary team meetings where staff and providers can debrief about challenging patients were beginning and were well-received.

Although our findings are specific to a safety-net HIV center, they are consistent with studies across healthcare systems, including other studies with HIV care providers,^14–20^ reporting that over half of physicians in the United States experience symptoms of burnout,^3,11,12^ due in part, to high healthcare system demands in general. The healthcare environment—with high patient volumes, limited time, and numerous administrative and clinical responsibilities—places physicians and other healthcare professionals in general at risk for burnout.^1–4^ This assessment revealed a necessity to enhance provider and staff self-care in a large HIV safety-net clinic and suggests that other similar HIV care settings (i.e. publicly funded HIV clinics) may also have a similar need because they serve similar patient populations with complex needs and face many of the same environmental constraints in their work settings. As a central tenant of TIC, self-care is an essential practice for all healthcare professionals (e.g. providers, nurses, and staff in all roles), especially those working with medically complex patients with trauma histories in low-resource environments.^30^ To promote well-being and prevent burnout of employees across various roles working in publicly funded HIV treatment settings specifically, and safety-net clinics generally, it is necessary for these clinics to provide more opportunities for employees to manage stress and emotional fatigue.

Adoption of TIC organizational strategies and service delivery practices in safety-net HIV clinics may not only help patients overcome traumatic experiences but adoption of specific workforce development strategies can also produce more effective providers and medical workforce, by reducing burnout, stress, emotional fatigue, and secondary trauma.^30^ Specifically, key TIC strategies to prevent secondary trauma and reduce stress and burnout include normalizing conversations about secondary trauma experiences at all levels of the organization to enhance the likelihood that staff will talk openly about it at meetings and to supervisors; implement clinical workload policies and practices that maintain reasonable standards for direct-care hours; increase the availability of opportunities for supportive professional relationships by promoting activities such as team meetings, peer supervision groups, staff retreats, and trainings focused on self-care; and provide opportunities for providers/staff to feel empowered within the organization through soliciting input on clinical and administrative policies that impact their work lives, such as workload and workforce development policies. Ultimately, the cost—to providers, staff, patients, clinics, and health systems—associated with burnout is high,^44^ and implementing provider and staff self-care practices can reduce medical errors and improve health outcomes among the most medically vulnerable populations.^3,5^ They may also be potentially cost saving to clinics and healthcare systems, though cost-effectiveness studies are lacking.

Other than TIC models which include strategies for reducing stress and secondary trauma experienced by staff and providers working with complex patient populations, evidence-based interventions to encourage provider and staff self-care in general are lacking. However, there are promising strategies for mitigating burnout.^2,5,6,45^ Current strategies and interventions are targeted toward individual staff and providers, medical care teams, and healthcare organizations. At the individual level, evidence highlights the importance of providers being responsible for their own health and wellness routines, such as exercising, eating healthy diets, and attending to their own medical care.^45^ Other strategies to reduce burnout among healthcare workers including taking deliberate steps to integrate their personal and professional lives,^45^ building support networks and connections with colleagues,^46,47^ and practicing self-awareness techniques (such as mindfulness, and cognitive behavioral techniques).^47,48^

On the organizational side, leadership can promote a culture shift within clinics, by supporting or making staff/providers aware of wellness initiatives,^47,48^ and creating spaces for peer interactions and support beyond “provider meetings.”^46,47^ As our results highlight, this was happening informally in the Center and administrators acknowledged how important this multi-disciplinary peer support was for staff/provider well-being and morale. Furthermore, creating intentional spaces to allow for peer support and multi-disciplinary interaction was perceived as feasible to implement in this resource-constrained setting as well. Importantly, these activities also cultivate collegiality and enhance employee satisfaction.^45^ Also important, but a bit more challenging to implement in resource-constrained settings are practices like setting reasonable productivity expectations,^5^ or utilize each team member’s competencies in meaningful ways, and allowing employees to operate at the “top of their license.”^45^ However, to the extent possible, leadership and supervisors could meet with employees individually to help them identify their strengths and afford them time to pursue their passions.^5^ In addition, organizations should devise strategies to reduce stigma associated with psychological issues among medical professionals.^5^ A 2008 national survey found that 1 out of 16 providers experienced suicidal ideation, but 60% of those individuals were reluctant to seek psychiatric assistance due to medical license concerns.^49^

Another promising strategy to reduce burnout involves maximizing the efficiency of medical teams, by taking work off the shoulders of providers and nurses and giving it to medical assistants or other support staff.^45^ By allowing office support staff to oversee clerical duties, 2 or 3 h of patient documentation and data entry per day is taken away from providers and nurses, allowing them to focus more time on patients.^5,50^ In one intervention, physicians were assigned multiple medical assistants to oversee clerical duties, which increased daily patient visits from 21 to 28, increased revenue from 20% to 30%, and improved satisfaction scores across all provider and staff categories.^48^ This is of particular importance in HIV safety-net clinics where patients often require time-consuming referrals to multiple support services (i.e. mental health, substance abuse, and social work). However, hiring additional staff may not be feasible for resources strapped safety-net healthcare settings.

### Limitations

Limitations of this study include the focus on a single HIV center. Also, not every employee was involved in our data collection, although we obtained data from ~34% of all employees of the center and we employed purposive sampling techniques for in-depth interviews to ensure diversity of perspectives in data. Despite these limitations, hundreds of similar publicly funded HIV centers operate across the United States,^51^ and this study suggests the need to enhance self-care services, especially services related to stress management, crisis/difficult patient debriefing, and trauma training in the context of publicly funded safety-net HIV care.

## Conclusion

At safety-net HIV treatment clinics, patients often have complex trauma histories along with other social and economic challenges, such as poverty, low literacy, and lack of insurance.^33,35^ Healthcare professionals working at safety-net clinics also often face additional stressors due to lack of essential supplies and resources, limited patient care space, and understaffing.^9,34^ Because these resource-poor environments place medical staff at risk for burnout,^9,34^ it may be particularly necessary for publicly funded HIV treatment centers that serve as long-term primary care homes to ensure staff and providers are receiving support and self-care services. Ultimately, even in resource-constrained environments, the adoption of organization-wide service delivery models, like TIC, in healthcare settings serving patients with complex needs (e.g. trauma histories) that prioritize provider and staff self-care and the adoption of self-care practices may lead to healthier providers and staff, thereby benefiting patient care and ultimately, patient health outcomes.
